# Case Report: A case of membranous nephropathy associated with primary Sjögren’s syndrome treated with telitacicept

**DOI:** 10.3389/fimmu.2025.1551094

**Published:** 2025-04-11

**Authors:** Juan Chen, Fangfang Zhou, Jian Shen, Jun Pang, Hanqing Chu

**Affiliations:** ^1^ Department of Nephrology, The Affiliated YangMing Hospital of NingBo University (Yuyao People’s Hospital), Ningbo, Zhejiang, China; ^2^ Department of Nephrology, Ningbo No.2 Hospital, Ningbo, Zhejiang, China

**Keywords:** primary Sjögren’s syndrome, membranous nephropathy, Telitacicept, B cells, urine protein, albumin

## Abstract

Primary Sjögren’s syndrome (pSS) is an autoimmune disease that often affects the exocrine glands. However, pSS can also affect the kidneys, most commonly involving the kidney interstitium. Recent studies have demonstrated that some membranous nephropathy (MN) cases are associated with Sjögren’s syndrome. However, unified recommendations for selecting immunosuppressive agents for treating MN associated with pSS are currently lacking. In the case reported herein, a patient with MN associated with pSS experienced improvement following methylprednisolone and telitacicept treatment.

## 1 Introduction

Primary Sjögren’s syndrome (pSS) is an autoimmune disease, and the prevalence of pSS with renal involvement ranges from 1% to 50% (1–3). There is a lack of uniform recommendations for the immunosuppressive treatment of membranous nephropathy (MN) associated with pSS. In patients with pSS, elevated serum B-cell activating factor (BAFF) and A proliferation-inducing ligand (APRIL) can be detected (4), and B-cell hyperactivity leads to renal injury in pSS patients (5). Telitacicept is a novel human transmembrane activator, calcium modulator, and cyclophilin ligand interactor-fragment crystallizable (TACI-Fc) fusion protein synthesized by recombinant deoxyribonucleic acid (DNA) technology. Compared with other B-cell inhibitors, telitacicept can affect B-cell differentiation at multiple sites by inhibiting the binding of BAFF and APRIL (6), and its efficacy and safety have been demonstrated in clinical studies on systemic lupus erythematosus (SLE) phase II and III, IgA Nephropathy (IgAN) phase II, and pSS phase II. In this case, the disease was controlled by methylprednisolone and telitacicept treatment (7–9). This case report presents the first use of telitacicept for treating MN associated with pSS. It includes details on the medication used during treatment and changes in serum albumin (ALB) and urinary protein levels during follow-up for clinical reference.

## 2 Case description

A 42-year-old female patient was admitted to the hospital in August 2023 with symptoms of dry mouth, dry eyes, and foamy urine lasting more than 5 months. The urinalysis conducted by the local health center revealed a urine protein level of 2+. Moreover, the patient was subsequently prescribed losartan potassium 25 mg orally once per day. As urine protein tests continued to be positive, tripterygium wilfordii tablets were added as part of the immunosuppressive treatment. One week before hospitalization, she visited the local health center again for treatment, and a repeat urinalysis revealed a urine protein level of 3+, positive urine occult blood, an ALB level of 26.8 g/L (40-55g/L), and a creatinine (Cr) level of 54 μmol/L (41-73μmol/L). The patient was then admitted to the outpatient department based on chronic nephritis syndrome for further treatment. The patient had a history of hypertension for more than 10 years, however, other personal and family histories were unremarkable. On physical examination, the patient had a body temperature of 37°C, pulse rate of 95 beats per minute, respiratory rate of 17 breaths per minute, blood pressure of 131/79 mmHg, height of 158 cm, weight of 72 kg, and Body Mass Index of 28.84 kg/m^2^. The patient was alert and her tongue was red. Although she had some glandular atrophy and corneal hyperemia, no skin erythema was observed. Clear breath sounds were noted in both lungs with no obvious dry or wet rales. She also had a regular heart rhythm with no obvious pathological murmurs, a flat and soft abdomen with no tenderness, no edema in either lower limb, and no tenderness in the joints and limbs. Further auxiliary examinations were performed after admission. Routine blood tests revealed the following results: white blood cell count of 4.2×10^9/^L (3.5-9.5×10^9/^L), platelet count of 167×10^9^/L (125-350×10^9^/L), and hemoglobin level of 131g/L (115-150g/L). Urinalysis revealed a pH of 6.0 (4.5-8.0), urine protein level of 2+ (negative), and negative results for the presence of red blood cells in the urine. The patient had a Cr level of 50 μmol/L (41-73μmol/L), ALB level of 23.4 g/L (40-55g/L), total cholesterol level of 5.29 mmol/L (0-5.20mmol/L), and erythrocyte sedimentation rate of 25 mm/h (0-20mm/h). The anti-phospholipase A2 receptor (PLA2R) antibodies level of 2.02 u/ml (<20 u/ml), a complete set of connective tissue tests was conducted, and the results were as follows: ANA 1:160-positive (negative), anti-SSA antibody-positive ++ (negative), anti-SSA antibody (anti-Ro-52 antibody) positive +++ (negative), negative for anti-ds-DNA antibody and anti-Sm antibody. The 24-hour urine protein quantification was 2939 mg/24h (28-141mg/24 h), immunoglobulin G (IgG) level of 13.10g/L (8.00-16.00 g/L), immunoglobulin A (IgA) level of 2.71g/L (0.70-3.30 g/L), immunoglobulin M (IgM)level of 0.71g/L (0.50-2.20 g/L), complement C3 level of 0.99g/L (0.83-1.77 g/L), complement C4 level of 0.33g/L (0.12-0.36 g/L), the patient Sjögren’s syndrome disease activity index (ESSDAI) scores is 15 (**Table 1**). Further, no abnormalities were identified in the patient’s rheumatoid factor, and the corresponding four domains in pre-transfusion testing. In addition, no abnormalities were detected on the electrocardiogram. On the lung computed tomography, tiny solid nodules of the lung imaging reporting and data system 2 (lung-RADS 2) were observed in the dorsal segment of the left lower lobe. Localized emphysema in the left upper lobe and calcification of the coronary artery wall were also discovered. Based on the urologic ultrasound, both kidneys were normal in size and shape, and the tear secretion test was positive for both eyes. A color Doppler ultrasound of the parotid and submandibular glands revealed diffuse lesions in the glands on both sides, but no abnormalities observed were in the ultrasound of the liver, gall bladder, spleen, and pancreas, as well as in the cardiac ultrasound.

**Table 1:** Characteristics of the patient before and after treatment.

Subject	Pre-treatment	After-treatment
symptoms	dry mouth, dry eyes, and proteinuria	no symptoms of dry mouth or dry eyes
Laboratory results		
IgG	13.10g/L	4.54 g/L
IgA	2.71g/L	0.64 g/L
IgM	0.71g/L	0.18 g/L
ALB	23.4 g/L	36.5 g/L
urine protein	2939 mg/24h	0.09 g/g.cr
ESSDAI score	15	0

IgG, immunoglobulin G; IgM, immunoglobulin M; IgA, immunoglobulin A; ALB, albumin; ESSDAI, Sjögren’s syndrome disease activity index.

Renal Pathology: The 21 glomerular lesions consisted of diffuse basement membrane thickening with segmental mesangial cells, mild hyperplasia of the mesangial matrix, and segmental endothelial cell proliferation. The small focal infiltration of lymphocytes, monocytes, and plasma cells (<25%). Three small spheres were detected in the immunofluorescence test, displaying diffuse granular staining with immunoglobulin G (IgG)1+++ (granular), IgG4++ (granular), and C1q++ (granular). The specimen tested negative for IgG2, IgG3, and the anti-PLA2R antibody. Electron microscopy demonstrated a mild thickening of the glomerular basement membrane of approximately 310-500 nm, with many granular electron-dense deposits under the epithelium. Additionally, mild hyperplasia of the mesangial segments with small electron-dense deposits was noted. Massive and electron-dense segmental subendothelial deposits were also observed.

**Figure 1:**
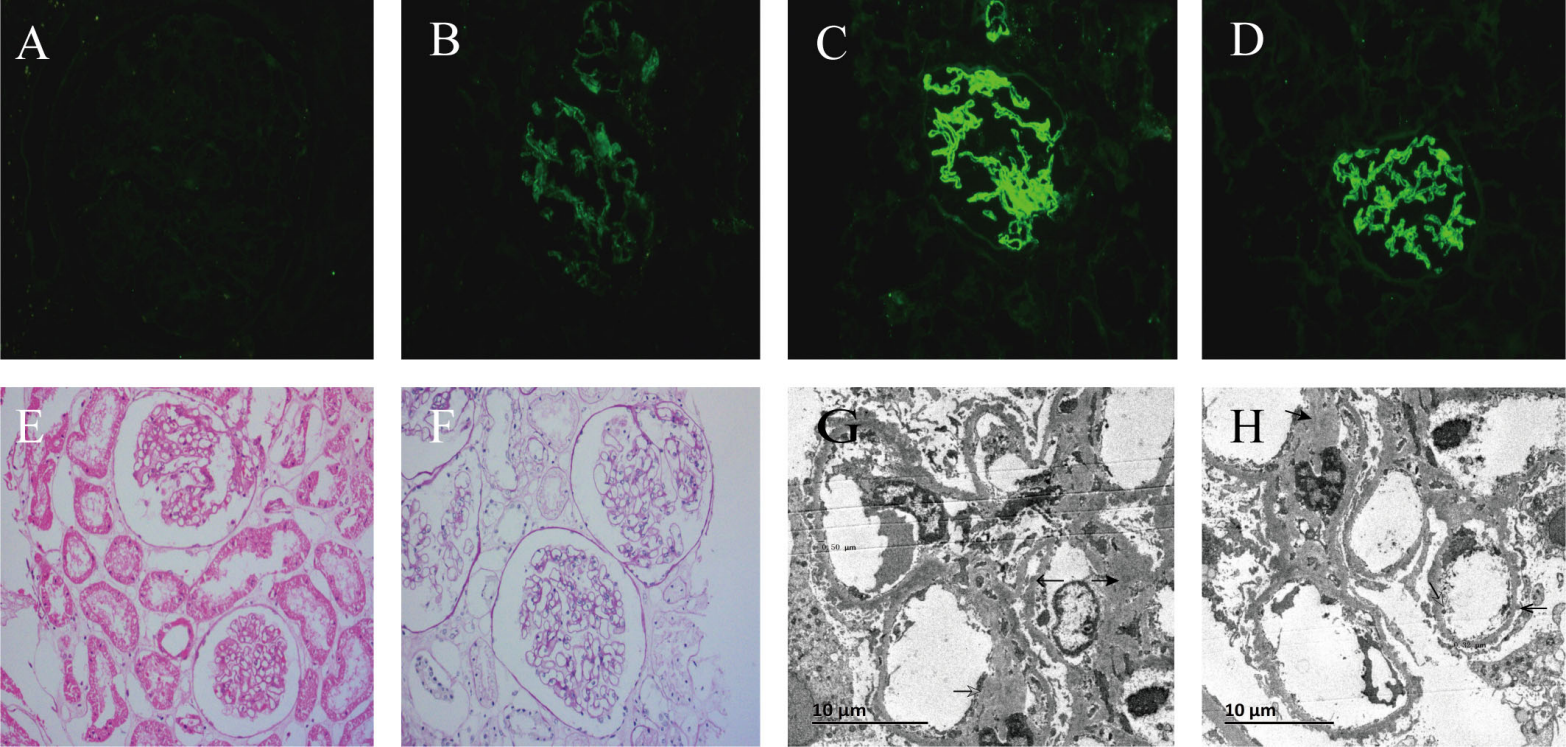
Immunofluorescence, light microscopy, and electron microscope of renal tissue from renal puncture biopsy. **(A)** is anti-phospholipase A2 receptor immunofluorescence (× 200);**(B)** is C1q immunofluorescence (× 200); **(C)** is immunoglobulin G (IgG) immunofluorescence (× 200); **(D)** is IgG1 immunofluorescence (× 200); **(E)** is hematoxylin and eosin staining (× 200); **(F)** is Periodic acid-Schiff staining (× 200); **(G)** is thickening of glomerular basement membrane (× 3000); **(H)** illustrates electron-dense material deposited of the mesangial segments, subendothelial and under the epithelium (×3000).

The patient’s renal biopsy demonstrated negative anti-PLA2R antibody staining, meanwhile, immunofluorescence revealed diffuse distribution of IgG1+++ and C1q++ staining (granular). Numerous granular electron-dense deposits under the glomerular epithelium were observed. Furthermore, massive electron-dense deposits were also observed under the mesangial endothelium, which was consistent with atypical membranous nephropathy (AMN). Combined with the clinical history and examination, the patient was considered to have MN associated with pSS (**Figure 1**).

**Figure 2:**
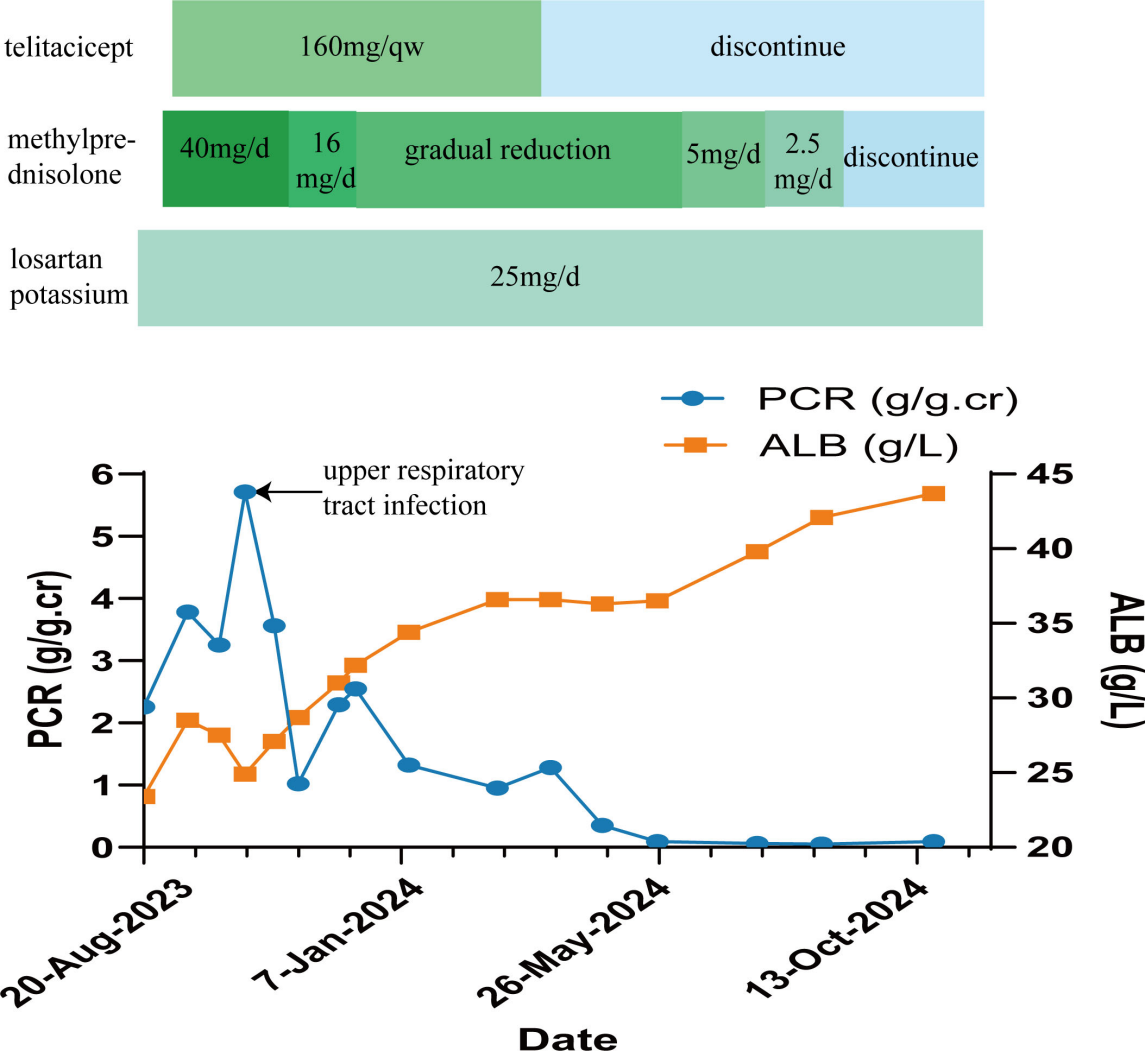
The changes in urinary protein and serum albumin (ALB) with medication during treatment.

Based on the aforementioned examinations and tests, the patient was diagnosed with pSS associated with MN, and hypertension stage 2. On September 14, 2023, she was administered a methylprednisolone 40 mg intravenous drip once per day, as part of the immunosuppressive treatment, and losartan potassium 25 mg orally once per day to lower blood pressure and urine protein levels. The patient also received other treatments such as calcium supplementation, stomach protectants, and lipid-lowering medicines. On September 18, 2023, immunosuppressive treatment with 160 mg of telitacicept was administered subcutaneously once per week (the course of telitacicept is six months). In October 2023, due to upper respiratory tract infection, the disease progressed to nephrotic syndrome, with the patient having a urine total protein-to-creatinine ratio (PCR) of 5.71 g/g.cr (0-0.20 g/g.cr) (**Figure 2**). ALB levels dropped to 24.9 g/L (40-55g/L) (**Figure 2**), and the Cr level was 62.8 μmol/L (41-73μmol/L). These values improved after anti-infective treatment. On November 13, 2023, the patient was re-evaluated. The PCR level was 1.02 g/g.cr (0-0.20 g/g.cr), and the ALB level was 28.7 g/L(40-55g/L). Given the improvement in the patient’s condition, the dosage of methylprednisolone was reduced to 16mg per day. This dosage was gradually decreased further based on the patient’s condition. On March 18, 2024, telitacicept was discontinued after six months of treatment. Throughout this period, the patient’s Cr levels normalized and stabilized. After eight months of treatment, the patient showed decreased levels of immunoglobulins: IgG at 4.54 g/L (8.00-16.00 g/L), IgA at 0.64 g/L (0.70-3.30 g/L), and IgM at 0.18 g/L (0.50-2.20 g/L). Additionally, urine protein tests returned negative, the patient achieved complete remission on May 25, 2024 (**Table 1**). Methylprednisolone was then discontinued in September 2024, while antihypertensive therapy with losartan potassium, 25 mg taken orally once daily, was maintained. Currently, the patient shows no symptoms of dry mouth or dry eyes, and her ALB and Cr levels are normal, with urine protein testing (**Figure 2**).

## 3 Discussion

pSS is an autoimmune inflammatory disease that primarily affects the exocrine glands, especially the salivary and lacrimal glands. The pathology of pSS is characterized by lymphocyte infiltration of the exocrine glands (10), and can be divided into primary and secondary types. The condition is more common in women, with an incidence of 6.92/100,000 person-years and a prevalence of 60.82/100,000 people (11). The main symptoms of pSS include dry mouth and eyes (5), but the condition is often accompanied by other symptoms, including fatigue and pain (12). Moreover, further involvement of the kidneys, lungs, liver, and heart can lead to corresponding clinical manifestations (5, 10). Here, the patient was a middle-aged female. According to the 2016 European League Against Rheumatism/American College of Rheumatology (EULAR/ACR) diagnostic criteria for pSS, which included positive serological anti-SSA antibodies, the patient had pre-onset symptoms of dry mouth and dry eyes, along with positive tear secretion tests in both eyes during the ocular examination, therefore the diagnosis of Sjögren’s syndrome was clear. Further, the patient had no history of head and neck radiotherapy, infectious diseases, and sarcoidosis, whereas amyloidosis and IgG4-related diseases were not considered. Antinuclear antibody assays were performed to exclude secondary Sjögren’s syndromes, such as SLE, thereby supporting the diagnosis of pSS.

Current literature reports the incidence of pSS combined with renal involvement ranges from 1% to 50%, reflecting the varying definitions of renal involvement (1–3). The main histological feature of pSS is chronic inflammation, characterized by diffuse or focal plasma lymphocyte infiltration (13, 14). Additionally, the condition affects the kidneys, with tubulointerstitial nephritis being the most common associated syndrome. Secondary glomerular damage has also been reported. Based on most studies, secondary glomerular damage in patients with pSS is believed to be mainly related to cryoglobulinemic membranoproliferative glomerulonephritis (13). In two recent large-scale studies based on renal biopsy pathologies, pSS combined with MN was considered the most common form of glomerulonephritis, with MN accounting for 36% and 36.9% (15, 16)of renal lesions in patients who underwent renal biopsy. This could be related to the combined influence of geographical factors, race, and other factors. Goules suggested that 15% of patients with pSS experience extra-epithelial complications mediated by immune complexes, which can manifest as glomerulonephritis. Moreover, this subgroup exhibited a poor prognosis and a high mortality rate due to an increased risk of lymphoma development (17).

Secondary MN accounts for approximately 20–25% (18) of MN cases, primarily occurring due to conditions such as hepatitis B, SLE, and Sjögren’s syndrome (19). In idiopathic MN, IgG4 often dominates or co-dominates (20) and is accompanied by high levels of anti-PLA2R antibodies (21), whereas the renal pathology of secondary MN is mainly characterized by IgG1, IgG2, and IgG3 deposition (18), with anti-PLA2R antibodies being negative. Serum anti-PLA2R antibodies have a high specificity for the detection of idiopathic MN, with positive tests in 75–85% of patients (18, 21). For the case reported here, the serum anti-PLA2R antibody test was negative, while the renal biopsy demonstrated positive results for IgG1 and C1q, confirming the absence of the anti-PLA2R antibody. Thus, the AMN diagnosis was supported considering the patient’s clinical symptoms (**Figure 1**). Excluding secondary factors such as hepatitis, SLE, rheumatoid arthritis, solid tumors, specific drugs, and toxins, this patient was considered to have a high likelihood of MN secondary to pSS.

At present, the pathogenesis of Sjögren’s syndrome remains unclear. Previous studies have demonstrated that B cells play an important role in the pathogenesis of pSS (22). Moreover, members of the tumor necrosis factor family, such as BAFF (also known as B lymphocyte stimulator (BlyS)) and APRIL, promote B cell differentiation, maturation, and plasma cell antibody production (23, 24). Some studies further suggest that elevated serum BAFF and APRIL levels can be detected in patients with pSS (4, 25). Furthermore, the proliferation of B lymphocytes in extra glandular epithelial cells has been confirmed. In addition, characteristic peri-epithelial lymphocyte infiltration, B cell overactivity, and other factors can cause renal damage in patients with pSS (5).

Treatment for MN associated with pSS mainly involves supportive symptomatic care, along with a combination of glucocorticoids and immunosuppressants. Some of these drugs are effective (26). Given the background of lymphoproliferation in patients with MN associated with pSS, the direct or indirect targeting of B cells has emerged as a new therapeutic modality (22). Moreover, clinical trials targeting B cells using treatments such as rituximab and belimumab are ongoing, with mixed reports of efficacy (27–31). Belimumab is a recombinant human IgG1-λ monoclonal antibody that inhibits the biological activity of soluble BLyS (32), discontinuation of belimumab has been reported to promote the recurrence of pSS (31). whereas rituximab is an anti-CD20 monoclonal antibody that depletes circulating B cells (33). Based on the current randomized controlled trial (RCT), there were no significant differences in pain, fatigue, or dry mouth with rituximab compared to placebo (34). Rituximab failed to reach its efficacy endpoints in two large randomized controlled studies (TEARS and TRACTISS) (35, 36). However, in a randomized, double-blind phase II clinical trial of patients with pSS, nearly complete CD20 B cell depletion was observed in the minor salivary glands and serum of patients sequentially treated with belimumab and rituximab, along with a significant decrease in ESSDAI scores (37). The poor efficacy of rituximab alone may stem from its limited impact on tissue B cells, as it primarily reduces CD20 B cells in the serum. In addition, the reactivity of BlyS in serum increases after treatment, which further promotes the differentiation and proliferation of B cells (14, 38).

In patients with pSS, BAFF and APRIL are overexpressed (4). Telitacicept, a novel human TACI-Fc fusion protein synthesized using recombinant DNA technology can affect the differentiation of B cells by binding to BAFF and APRIL (6). The drug was launched in China in March 2021 (39). As opposed to other B cell targeting drugs, telitacicept has two functional mechanisms. Not only does telitacicept inhibit the binding of BAFF to arrest the development of immature B cells, but it can also block APRIL, thereby preventing mature B cells from continuing their differentiation into plasma cells. Therefore, inhibiting the survival of plasma cells, and reducing the release of antibodies (40). The efficacy and safety of telitacicept have been confirmed in phase II clinical studies of SLE, IgAN, and pSS phase II studies (7–9). In a 52-week randomized, double-blind, placebo-controlled phase III trial of telitacicept in the treatment of SLE (National Clinical Trial 04082416), the systemic lupus erythematosus responder index (SRI)-4 response rate was 82.6% in the telitacicept group compared with 38.1% in the placebo group, which is a better efficacy than the placebo group (6, 41). In another retrospective, multicentre study, 72 patients with active SLE were treated with telitacicept. After 24 weeks of telitacicept treatment, the mean systemic lupus erythematosus disease activity index 2000 (SLEDAI-2K) score decreased from 9.88 to 4.43. After 52 weeks of telitacicept treatment in patients with lupus nephritis, median 24-hour urine protein decreased from 1323.5 mg to 224.0 mg. Of the 9 patients with lupus nephritis, 7 patients achieved partial remission and 6 patients achieved complete remission. The proportion of patients with infections in the telitacicept group was 23.6%. Overall, no serious adverse events occurred during the treatment of SLE (6, 42). In a randomized, double-blind, placebo-controlled, multicenter phase II clinical trial based on patients with pSS, the administration of telitacicept at a dose of 160 mg significantly reduced patient ESSDAI scores, the Multidimensional Fatigue Inventory (MFI)-20, and serum immunoglobulin IgG, IgA, and IgM levels, as compared to levels in those with the placebo (p < 0.05) (8). In terms of safety, the main adverse reactions were local injection site reactions. In the telitacicept 240 mg group, two patients experienced serious adverse events (Common Terminology Criteria for Adverse Event (CTCAE) grade 3)) in the form of acute pyelonephritis and leukopenia. The acute pyelonephritis resolved after discontinuation of telitacicept and antibiotic therapy, while the leukopenia persisted. No CTCAE grade 4 or 5 adverse events were reported. In addition, no increased risk of infection was observed in the telitacicept group compared with the placebo group (8, 34). Concerning the case reported here, she was in complete remission of treatment with telitacicept and methylprednisolone. Moreover, after 6 months of follow-up, the patient currently has no obvious symptoms of dry mouth and dry eyes, with normal albumin levels. Additionally, urine protein is now negative (**Figure 2**).

This study comprises a case of MN associated with pSS involving treatment with telitacicept, which was demonstrated to be effective. As opposed to other B cell inhibitors, telitacicept can inhibit the binding of BlyS and APRIL, affect the differentiation of B cells at multiple sites, and effectively control the condition of MN associated with pSS. As this was an individual case report, more clinical studies are needed to confirm the efficacy of telitacicept for the treatment of pSS and MN associated with pSS.

## Data Availability

The raw data supporting the conclusions of this article will be made available by the authors, without undue reservation.
