# Supplementary material for: Case Report: A case of membranous nephropathy associated with primary Sjögren’s syndrome treated with telitacicept
Source: Front Immunol. 2025 Apr 11;16:1551094. doi: 10.3389/fimmu.2025.1551094 (PMC12021926; doi:10.3389/fimmu.2025.1551094)
Supplement: Supplementary file 1 [file Table1.docx]

| subject | pre-treatment | after-treatment |
| --- | --- | --- |
| symptoms | dry mouth, dry eyes,  and proteinuria | no symptoms of dry mouth  or dry eyes |
| laboratory results |  |  |
| IgG | 13.10g/L | 4.54 g/L |
| IgA | 2.71g/L | 0.64 g/L |
| IgM | 0.71g/L | 0.18 g/L |
| ALB | 23.4 g/L | 36.5 g/L |
| urine protein | 2939 mg/24h | 0.09 g/g.cr |
| ESSDAI score | 15 | 0 |
